# A Tutorial on Hardware-Implemented Fault Injection and Online Fault Diagnosis for High-Speed Trains

**DOI:** 10.3390/s21175957

**Published:** 2021-09-05

**Authors:** Xiaoyue Yang, Xinyu Qiao, Chao Cheng, Kai Zhong, Hongtian Chen

**Affiliations:** 1 School of Rail Transportation, Wuyi University, Jiangmen 529020, China; xyyeoh@outlook.com; 2 School of Astronautics, Harbin Institute of Technology, Harbin 150001, China; qiaoxinyu2018@126.com; 3 School of Computer Science and Engineering, Changchun University of Technology, Changchun 130012, China; chengchao@ccut.edu.cn; 4 Institutes of Physical Science and Information Technology, Anhui University, Hefei 230601, China; kaizhong0402@ahu.edu.cn; 5 Department of Chemical and Materials Engineering, University of Alberta, Edmonton, AB T6G 1H9, Canada

**Keywords:** fault injection (FI), fault diagnosis (FD), hardware-implemented (HIL) platforms, electrical drive systems, high-speed trains

## Abstract

Electrical drive systems are the core of high-speed trains, providing energy transmission from electric power to traction force. Therefore, their safety and reliability topics are always active in practice. Among the current research, fault injection (FI) and fault diagnosis (FD) are representative techniques, where FI is an important way to recur faults, and FD ensures the recurring faults can be successfully detected as soon as possible. In this paper, a tutorial on a hardware-implemented (HIL) platform that blends FI and FD techniques is given for electrical drive systems of high-speed trains. The main contributions of this work are fourfold: (1) An HIL platform is elaborated for realistic simulation of faults, which provides the test and verification environment for FD tasks. (2) Basics of both the static and dynamic FD methods are reviewed, whose purpose is to guide the engineers and researchers. (3) Multiple performance indexes are defined for comprehensively evaluating the FD approaches from the application viewpoints. (4) It is an integrated platform making the FI and FD work together. Finally, a summary of FD research based on the HIL platform is made.

## 1. Introduction

Over the past five decades, the rapid development of high-speed trains relying on multiple electrical traction units has been witnessed [1,2,3]. Nowadays, high-speed trains have become one of the most important transportation means thanks to their inherent advantages such as rapidity, comfort, and high efficiency [4]. However, unexpected railway accidents usually happen all over the word because of external and internal factors [5,6,7]; for example, design defects of high-speed trains and limits of usage life will induce the faults appearing in trains and may further lead to catastrophic accidents [8,9,10]. To the best of our knowledge, electrical drive systems can be viewed as the heart of high-speed trains, whose reliability and safety are crucially important to ensure the whole train operates safely [5]. Therefore, fault diagnosis (FD) for electrical drive systems is always an active topic from theoretical and practical researchers in the transportation field [6].

In order to investigate FD issues for electrical drive systems of high-speed trains, two key steps involved are: (1) ***fault injection*** (FI) which can replicate faults in a true manner [11], and (2) ***FD*** which can successfully and rapidly detect and diagnosis faults [5]. The first step can simulate various faults appearing in electrical drive systems on the one hand and provide sufficient data sets for fault analysis and experimental verification on the other hand [12]. Based on first principles or collected data sets from high-speed trains, to design reasonable and effective algorithms is the second step for online FD purposes [5].

Initially applied to the centralized systems, especially dedicated fault-tolerant computer architectures in the early 1970s, FI is widely used to evaluate the fault-tolerant performance and injects the artificial faults into the system of interest [13]. The FI design can speed up the occurrence of faults to observe the system changes and also provides a way for analyzing the efficiency of new fault FD mechanisms [14]. Up to now, there are three major categories of FI techniques: hardware-based, software-based and emulation-based [15]. The hardware-based FI method involving the extra hardware, which is specially designed, allows injecting faults into the target system. This technique works efficiently for large-scale-integration circuits but meanwhile introduces a high risk of damage for the target system. The software-based method is targeted to applications in operating systems, and it cannot inject faults into the inaccessible location of software. Unlike the former two methods, the detailed simulation model is crucial for the emulation-based method, which leads to a maximum amount of observability and controllability, whereas model development is time-consuming and inaccurate. These FI methods mainly concentrate on evaluating the dependability of microelectronics systems or software programs [16]. Nevertheless, apart from the microelectronics system, a traction control system (TCS) consists of the power electronic and mechanical parts [17].

Nowadays, the hardware-in-the-loop (HIL) technique is widely applied to the power system field, which requires the entire system to be modelled inside the real-time simulator and does not involve external interfacing or input/output. In fact, the primary goal of HIL simulation is testing of the physical controller (including the hardware design and control strategy). A real-time simulator needs to solve the model equations for a one-time step within the same time in a real-world clock. If the execution time for the simulation of the system is shorter than or equal to the selected time-step, the simulation is considered to be real-time. The study [18] presents an HIL platform to simulate the open-switch fault of one rectifier in TCS, which aims to verify the proposed FD method. In [12], a multiprocessor HIL-based FI is proposed for real-time simulation of faults in traction control systems, and a timing optimization method is proposed to deal with overruns induced by real-time FI.

Generally, the current FD methods for electrical drive systems of high-speed trains can be definitely divided into three categories: signal analysis-based, model-based and data-driven methods [5,19]. For signal analysis-based strategies, the difference between nominal and fault statuses reflected in time domain [20], frequency domain [21] and time-frequency domain [22], are desirable features used for the FD purposes. As pointed out in [6], the commonly used FD method in practice is the so-called human inspection, which belongs to signal analysis-based methods. Based on a well-established system model of electrical drive systems, model-based methods can effectively perform FD tasks via designing residual generators [23]. Most recently, data-driven FD methods for electrical drive systems of high-speed trains have been intensively developed and widely accepted because of their simplicity of design and ease of implementation [24].

In the previous studies, FI and FD are independently and separately designed, which poses inevitable difficulties in the validation of online FD algorithms. Motivated by the aforementioned observations, the main contributions of this paper are summarized as follows:

1. Introduce an integrated validation platform where FI and FD can work together in real time.
2. Base an evaluation system for FD systems where various performance indexes are defined.
3. Review the data-driven FD literature whose verification is based the designed HIL platform.

The rest of this study is organized as follows. Section 2 describes the preliminaries of electrical drive systems, typical fault types, and the objective pursued in this paper. Section 3 details the FI methodologies using hardware-implemented manners. Based on the existing FD research, Section 4 summarizes the FD algorithms together with implementation procedures. Section 5 concludes this paper with future work and promising research directions.

## 2. Background

In this section, preliminaries of electrical driven systems, associated with various faults, will be described, followed by the objectives this work is dedicated to.

### 2.1. Electrical Drive Systems of High-Speed Trains

**Figure 1:**
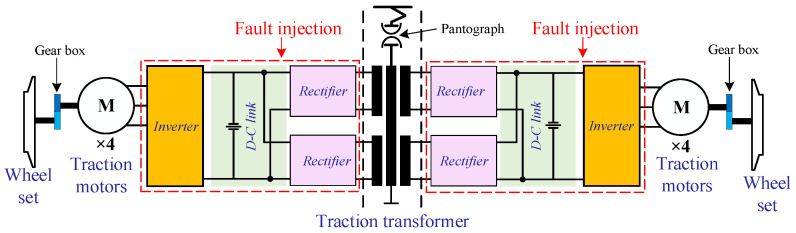
The schematic diagram of electrical drive systems in high-speed trains [24].

In this study, the CRH2A-type high-speed train is taken into consideration in which four traction systems provide traction power of the entire train [4]. As presented in [2], the traction system is one core unit consisting of a three-level source inverter (VSI), four induction motors, a traction control unit (TCU), filters, etc. Its control strategy is space vector pulse-width-modulation (SVPWM), and the corresponding schematic diagram is depicted in Figure 1.

In the traction system of CRH2A-type high-speed trains, six sensors are equipped to collect real-time observations that will be used as the input of both the double proportional integral (PI) controller and the supervision unit. After setting a given traction speed, TCU can achieve expected performance by adjusting gate control signals of VSI based on the SVPWM strategy. By comparing online samplings and the corresponding pre-designed thresholds, the supervision unit adopted in the existing high-speed trains may not be effective for successful detection of parts’ faults, and then an auto-protecting mode will be activated [2]. Actually, either high missing alarm ratios (MARs) or high false alarm ratios (FARs) in traction systems of high-speed trains will lead to unsatisfactory FDD results and should be prohibitive. An acceptable trade off between MAR and FAR should be at least achieved for FDD in traction systems of high-speed trains.

### 2.2. Fault Types

For the electrical drive systems of high-speed trains, there are different ways to judge which category one fault belongs to. For example, fault types can be determined depending upon fault amplitude [24], fault location [11] and fault-duration time [25].

According to the fault location, faults in electrical drive systems could be divided into into the following four scenarios:

1. Sensor faults: Faults may happen in voltage sensors, current sensors, speed sensors, temperature sensors, etc.
2. Converter faults: Aging components such as performance degradation of capacitance, short- and open-circuit of insulated-gate bipolar transistors (IGBTs) are common faults appearing in traction converters.
3. Motor faults: Rotor-broken bar, air-gap eccentricity, together with interturn-short circuits will induce faults in traction motors.
4. Control-unit faults: Errors in both analog and digital signals are responsible for faults in traction control units.

According to the appearance time of faults, two categories are summarized as follows:

1. Permanent faults: Some hardware malfunctions such as open circuit of IGBT and gear war belong to the permanent faults.
2. Intermittent/Transient faults: These faults appear, disappear and reappear nondeterministically, and the duration time is short such that important features are difficult to be captured [26].

In addition, faults in electrical drive systems can be categorized into three types based on their amplitudes:

1. Incipient faults: This type of fault is usually characterized by small amplitudes, tiny influences and common faults as time goes on [24]. These faults in electrical drive systems of high-speed trains are, for example, sensor faults and aging components.
2. Common faults: There are some kinds of faults that have larger amplitudes than incipient faults and at the same time affect the performance of trains in a considerable means. Timely maintenance is necessary when they occur.
3. Failures: The failure means malfunctions of components or systems. It usually results in system performance far from the acceptable operation. The broken IGBT will distort three-phase currents, causing degraded traction efficiency.

### 2.3. Objectives

Based on preliminaries of electrical driven systems and fault types [27], the proposed HIL platform can simulate a variety of failure scenarios in electrical drive systems of high-speed trains. It can effectively avoid the following problems: (1) Design the extra hardware in hardware-based fault injection technology; (2) Real-time debugging difficulty in software-based fault injection technology. It fully provides the experimental verification convenience for peers. It contributes to the objective and comprehensive evaluation of the proposed fault diagnosis algorithm. At the same time, it is possible to transform the research results into practical applications or give technical guidance for engineers.

## 3. Fault Injection Methodology

### 3.1. Signal-Based Fault Injection Methods

As mentioned in Section 2, among the four main components of traction systems, there are some typical electronic-subsystems and mechanical equipment, such as IGBT in converters, monitoring or communication modules in TCU and rotors in traction motors. These subsystems maintain the stable operation of TCS, while they may also malfunction. Some faults are attributed to inaccuracy during the development, while others can originate from external causes such as production process defects or environmental stress (including heating, electromagnetic, mechanical stress, etc.). Once fault occurs, the electrical characteristics of faulty components are distinct from the normal status, and these changes are directly reflected in the output signals.

As mentioned in Section 2, the fault scenarios can be described qualitatively regarding the measurements. In order to generate the artificial fault scenarios, the signal-based fault injection method is introduced, providing the observable signals for FD as well as the associated fault prognosis.

For a faulty component or subsystem, its *D* fault types can be written as:

(1) $$f=\{f_{1},\cdots ,f_{i},\cdots ,f_{D}\}$$

where i=1,⋯,D. According to the fault $f_{i}$ of consideration, a set of corresponding signals will be generated. Based on the characteristics such as time and amplitude, the fault signal f(t) can be defined as follows:

(1) If f(t) is the transient fault, then
  (2) $$f\left(t\right)=\varepsilon \left(t-t_{s}\right)-\varepsilon \left(t-t_{e}\right)\cdot \lambda \left\{a_{j}\right\}.$$
(2) If f(t) is the intermittent fault, then
  (3) $$f\left(t\right)=\sum_{j=1}^{N}\sum_{j=1}^{n_{j}}\left[\varepsilon \left(t-\left(T_{j+1}+\tau_{j}\cdot \rho_{j}\right)-T_{j}-\varepsilon \left(t-T_{j+1}\right)\right)\right]\cdot \lambda \left\{a_{j}\right\}.$$
(3) If f(t) is the permanent fault, then
  (4) $$f\left(t\right)=\varepsilon \left(t-t_{s}\right)\cdot \lambda \left\{a_{j}\right\}.$$

From (2) to (4), ε signifies the step function; $t_{s}$ is the activation time of faults; λ{·} represents the threshold function for selecting analog or digital signals; $a_{j}$ means the amplitude of impulses. For the intermittent-fault cases, *N* is the total number of impulse-sequence types; *j* represents the index of the category of impulse sequences; $n_{j}$ means the number of pulses; $\tau_{j}$ denotes the period of the *j*-th impulse sequence; ρ is the duty cycle of impulse sequences. In fact, (2)–(4) cover a spread spectrum that can describe transient faults, intermittent faults and permanent faults.

**Figure 2:**
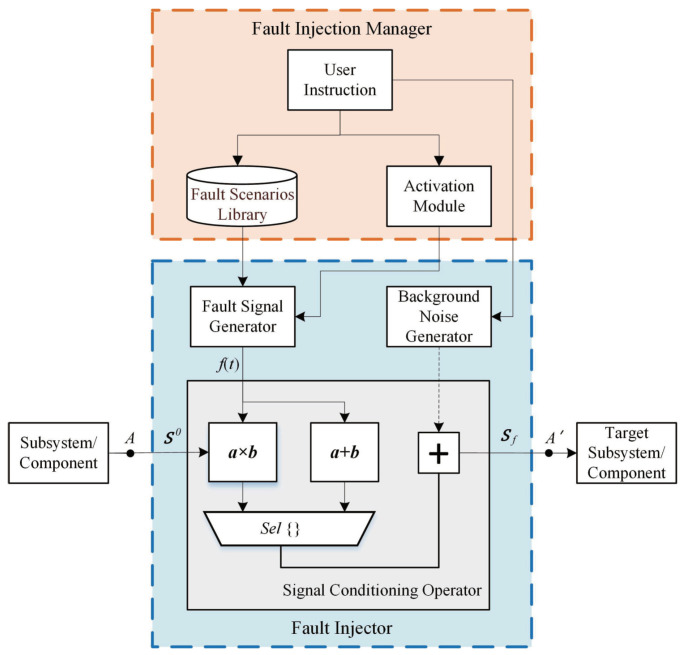
The framework of the signal-based FI method.

Along with the aforementioned descriptions, the framework of the signal-based FI method is shown in Figure 2, where the FI manager controls the injection process. First, the fault scenarios of the tested subsystem or components are analyzed, and the FI point *A* is determined. Hence, the original signal $S^{0}$ will be obtained. Based on the fault types (which will be injected into the subsystem) confirmed by user instructions, the activation module then provides the activation time of the faults. After that, the fault signal *f* described by (2)–(4) is determined by the fault library. By this means, the fault injection signal $S_{f}$ can be obtained by the signal conditioning operator. Specifically, the signal conditioning operator consists of three kinds of operators (i.e., the adder, multiplier and multiplexer). Because subsystems/components are connected by the wires, the FI point can be split into *A* and $A^{\prime }$. As depicted in Figure 2, the FI is placed between the real and target subsystems. Consequently, the input signal of the target subsystems/components is replaced by the FI signal, and thereby the signal-based FI targets can be implemented.

### 3.2. Hardware-in-the-Loop Fault Injection for Traction Systems

For the purpose of realizing the HIL fault injection, this work takes into consideration the following aspects:

(1) A real-time simulation of models consumes a large amount of FPGA resources, especially for power electronics-based apparatus. However, not all models are required for such a short execution time.
(2) High FPGA resources will be consumed when the FI signals are inserted.

**Figure 3:**
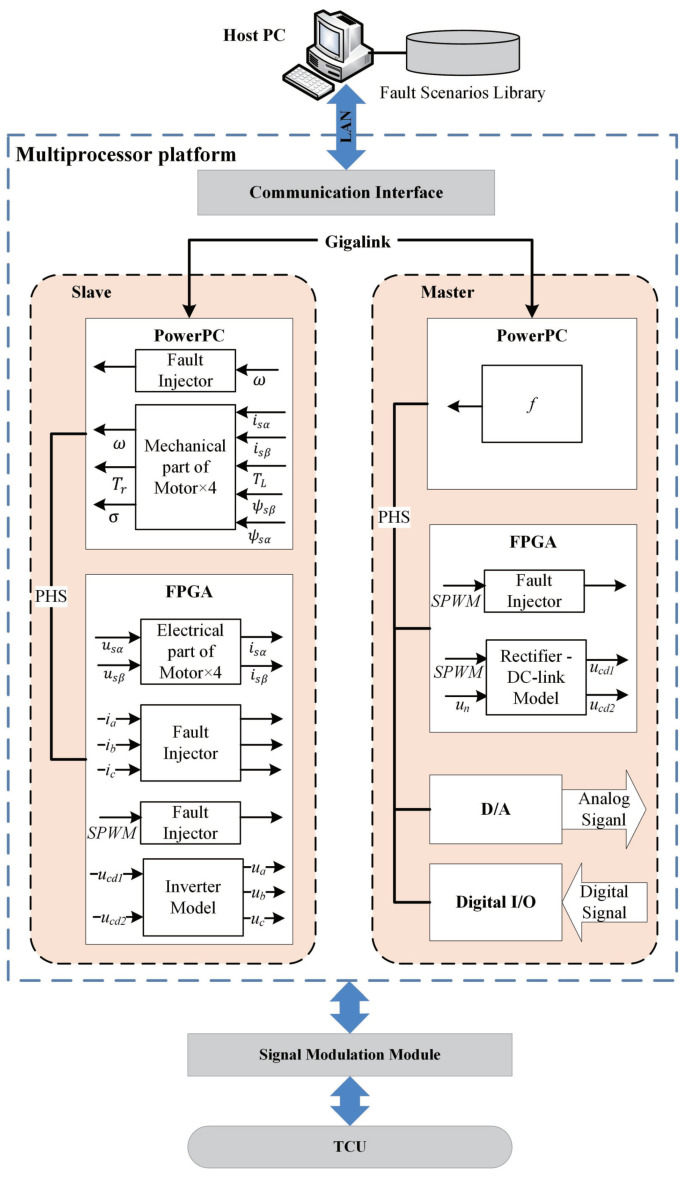
Schematic views of multi-FPGAs/CPUs HIL platform for real-time fault injection.

Based on a multiprocessor structure, the work will present an HIL fault injection platform for electrical drive systems with consideration of the two above mentioned aspects. As shown in Figure 3, the HIL-based FI structure consists of a master-slave system based on multi-FPGAs/CPUs, physical TCU, signal modulation module, host PC, fault scenarios library, D/A and digital I/Os, Gigalink and peripheral high-speed bus (PHS). Besides, the variable transmission between FPGA and CPU is implemented by the PHS bus.

## 4. Fault Diagnosis Methodology

This section will first develop the fault detection strategies including how to extract the fault features and how to establish the test statistics. Second, by the use of these fault features, several fault diagnosis strategies will be formulated. Based on the results including fault detection and FD, a comprehensive evaluation index will be obtained.

### 4.1. Fault Detection

Let the number of all measurement variables *z* be $k_{z}$. When a fault $f_{i}\left(k\right)$ appears in electrical drive systems, unexpected deviations will be reflected in *z* such that

(5) $$z^{f}\left(k\right)=z\left(k\right)+\Omega_{i}f_{i}\left(k\right)$$

where *k* represents the sampling step, $\Omega_{i}$ is the matrix signifying the fault direction, $∥f_{i}∥$ describes the deviational magnitude caused by $f_{i}$ and the subscript “*f*” means different faulty conditions [24].

In (5), *z* reflects the uncertainty caused by the surrounding noises and external disturbances that are unknown beforehand in practical applications. Whilst, Ωf results in unexpected deviations on *z*.

For the fault detection purpose, we consider the high-speed trains to work under a steady condition, i.e., at a given traction speed. Furthermore, the moving-window technique is used for enlarging the fault features. Due to the simplicity, the stacked data in a certain size of the moving window is defined as its original form. To detect the fault occurring in electrical drive systems, two necessary steps are:

(1) To extract fault features that are helpful for addressing high-frequency (online) data.
(2) To define a test statistic, based on which a reliable detection result of faults can be returned.

#### 4.1.1. Data-Driven Feature Extraction

Some important features are hidden in the measurement signals, as summarized in Table 1. Among them, the mean, covariance and slope are employed in our designed platform to detect and diagnose different faults. Remarkably, the slope is an important index that can be utilized for determining incipient faults.

For achieving online fault detection tasks where both sampling frequency and dimension of signals are high, principal component analysis is used for improving computation efficiency. Therefore, z(k) on the principal and residual subspace become

(6) $$\begin{array}{c} \hat{z}\left(k\right)=P_{p}P_{p}^{T}z\left(k\right)\\ \tilde{z}\left(k\right)=P_{r}P_{r}^{T}z\left(k\right) \end{array}$$

where $\hat{z}\left(k\right)$ and $\tilde{z}\left(k\right)$ are respectively principal and residual components of z(k); $P_{p}\in R^{k_{z}\times k_{p}}$ and $P_{r}\in R^{k_{z}\times \left(k_{z}-k_{p}\right)}$ are the loading matrices which are obtained via

(7) $$E\left({\left(z-E\left(z\right)\right)}^{2}\right)=\left[P_{p}\;P_{r}\right]\left[\begin{array}{cc} \Lambda_{p} & 0\\ 0 & \Lambda_{r} \end{array}\right]{\left[P_{p}\;P_{r}\right]}^{T}.$$

In addition, $k_{p}$ represents the number of principal components and satisfies $k_{p}\ll k_{z}$. The information of measurement variable z(k) is reflected in the variance. It should be noted that z(k) obeys Gaussian distribution. As mentioned in [24,28,29], the operation of traction systems is accompanied by non-Gaussian signals. In order to deal with the measured non-Gaussian signals, independent component analysis (ICA) is adopted in FDD of traction systems. Therefore, the hidden statistically independent components can be extracted by

(8) $$\begin{array}{c} \hat{s}\left(k\right)=Wz\left(k\right)\\ s.t.\;\max\;M(\hat{s}\left(k\right)) \end{array}$$

where $\hat{s}\left(k\right)$ is the estimation of independent components; M is the non-Gaussian measurement function, whose function is detailed in [30].

Different from the PCA and ICA approaches, canonical correlation analysis (CCA) is to find the maximum correlation between system input and output. Then, canonical variables are extracted among measured signals. Suppose that the model has input vector $u_{o}\in R^{l}$ and output vector $y_{o}\in R^{m}$ such that

(9) $$\left[\begin{array}{c} u_{o}\\ y_{o} \end{array}\right]∼N\left(\left[\begin{array}{c} u_{u}\\ u_{y} \end{array}\right],\left[\begin{array}{cc} \Sigma_{u} & \Sigma_{uy}\\ {\Sigma^{T}}_{uy} & \Sigma_{y} \end{array}\right]\right)$$

where Σ is covariance matrices. Then, the correlation evaluation is defined as

(10) $$\begin{array}{c} \Xi =\Sigma_{u}^{-1/2}\Sigma_{uy}\Sigma_{y}^{-1/2}. \end{array}$$

Based on an SVD on Ξ, the matrix Ξ can be decomposed as

(11) $$\begin{array}{c} \Xi =\Gamma \Sigma R^{T} \end{array}$$

with $\Gamma =(\gamma_{1},\ldots ,\gamma_{l})$, $\Sigma =\left[\begin{array}{cc} \Sigma_{\kappa } & 0\\ 0 & 0 \end{array}\right]$, $R=(r_{1},\ldots ,r_{m})$. where κ is the number of principal components, $\Sigma_{\kappa }$ is canonical correlation matrix [31]. Then, the canonical correlation vectors are given as

(12) $$\begin{array}{c} J=\Sigma_{u}^{-1/2}\Gamma \in R^{ll}\\ L=\Sigma_{y}^{-1/2}R\in R^{mm} \end{array}$$

Obviously, CCA method maintains the following properties [32]

(13) $$\begin{array}{c} \Gamma \Gamma^{T}=I_{k_{zu}},\;R^{T}R=I_{k_{yy}},\;J^{T}\Sigma_{uy}L=\Sigma L^{T},\\ \Sigma_{uy}^{T}=\Sigma^{T}J^{T}\Sigma_{u},\;J^{T}\Sigma_{y}=\Sigma L^{T}\Sigma_{y}. \end{array}$$

The three methods mentioned above have shown superior performance in static FDD. However, as the system state (such as the controller adjust the operation), the data will have dynamic characteristics [33]. Therefore, dynamic fault diagnosis methods have been widely used in high-speed trains [34]. The state space model of the traction system can be defined as [6]:

(14) $$\begin{array}{c} x(k+1)=Ax(k)+Bu(k)+w(k)\\ y(k)=Cx(k)+Du(k)+v(k) \end{array}$$

where $x\in R^{m_{x}}$ is the state of electrical drive systems. In general, $w\in R^{m_{x}}$ and $v\in R^{m_{y}}$ are noise sequences and are normally distributed. Matrices *A*, *B*, *C*, *D* are appropriately dimensioned in electrical drive systems. Specifically, the matrices are the change in angular velocity at different times. For example, the matrice *A* is given as

(15) $$\begin{array}{c} A=\left[\begin{array}{cccc} \frac{-R_{r}L_{m}^{2}-R_{s}L_{r}^{2}}{\sigma L_{s}L_{r}^{2}} & 0 & \frac{R_{r}L_{m}}{\sigma L_{s}L_{r}^{2}} & \frac{L_{m}\omega_{r}}{\sigma L_{s}}\\ 0 & \frac{-R_{r}L_{m}^{2}-R_{s}L_{r}^{2}}{\sigma L_{s}L_{r}^{2}} & -\frac{L_{m}\omega_{r}}{\sigma L_{s}} & \frac{R_{r}L_{m}}{\sigma L_{s}L_{r}^{2}}\\ \frac{L_{m}R_{r}}{L_{r}} & 0 & -\frac{R_{r}}{L_{r}} & -\omega_{r}\\ 0 & \frac{L_{m}R_{r}}{L_{r}} & -\omega_{r} & -\frac{R_{r}}{L_{r}} \end{array}\right], \end{array}$$

where $\omega_{r}$ is the motor rotor speed, and σ is the coefficient of magnetic leakage which can be obtained by $\sigma =(L_{s}L_{r}-L_{m}^{2}/\left(L_{s}L_{r}\right))$. $L_{s}$, $L_{r}$ and $L_{m}$ are inductance in stator side, inductance in rotor side and mutual inductance of motor, respectively. $R_{r}$ and $R_{s}$ are resistance in stator side and in rotor side, respectively. Obviously, matrix *A* shows that parameters change in real time. Therefore, it is significant to adopt dynamic methods for diagnosing faults in electrical drive systems.

Next, the following three notations are introduced to describe electric drive systems of high-speed trains:

(16) $$\begin{array}{c} w_{s}\left(k\right)=\left[w^{T}\left(k-s\right)\cdots w^{T}\left(k\right)\right]\in R^{(s+1)n}\\ \Omega_{k}=\left[w(k)\cdots w(k+N-1)\right]\in R^{n\times N}\\ \Omega_{k,s}=\left[w(k)\cdots w(k+N-1)\right]={\left[\Omega_{k-s}\cdots \Omega_{k}\right]}^{T}\in R^{(s+1)n\times N} \end{array}$$

where *s* and *N* are some integers.

Based on the parity space approach, the state space model (14) of electric drive systems is re-written as

(17) $$\begin{array}{c} Y_{k,s}=\Gamma_{s}X_{k-s}+H_{u,s}U_{k,s}+H_{w,s}W_{k,s}+V_{k,s}\in R^{\left(s+1\right)m_{y}\times N}, \end{array}$$

(18) $$\begin{array}{c} X_{k-s}=\left[x(k-s)\cdots x(k-s+N-1)\right],\Gamma_{s}={\left[C^{T}{\left(CA\right)}^{T}\cdots {\left(CA^{s}\right)}^{T}\right]}^{T},\\ H_{u,s}=\left[\begin{array}{cccc} D & 0 & \cdots & 0\\ CB & \ddots & \ddots & 0\\ \vdots & \ddots & \ddots & \vdots \\ CA^{s-1}B & \cdots & CB & D \end{array}\right],H_{w,s}=\left[\begin{array}{cccc} D & 0 & \cdots & 0\\ C & D & \ddots & \vdots \\ \vdots & \ddots & D & 0\\ CA^{s-1} & \cdots & C & D \end{array}\right], \end{array}$$

where $\Gamma_{s}\in R^{\left(s+1\right)m_{y}\times m_{x}}$, $H_{u,s}\in R^{\left(s+1\right)m_{y}\times \left(s+1\right)m_{u}}$, and $H_{w,s}\in R^{\left(s+1\right)m_{y}\times \left(s+1\right)m_{u}}$. $H_{w,s}W_{k,s}+V_{k,s}$ represents the noise of electric drive systems.

The state space model (17) can be further written into

(19) $$\begin{array}{c} \left[\begin{array}{c} U_{k,s}\\ Y_{k,s} \end{array}\right]=\left[\begin{array}{cc} I & 0\\ H_{u,s} & \Gamma_{s} \end{array}\right]\left[\begin{array}{c} U_{k,s}\\ X_{k-s} \end{array}\right]+\left[\begin{array}{c} 0\\ H_{w,s}W_{k,s}+V_{k,s} \end{array}\right]. \end{array}$$

Construct

(20) $$\begin{array}{c} Z_{f}=\left[\begin{array}{c} U_{k,s}\\ Y_{k,s} \end{array}\right],Z_{p}=\left[\begin{array}{c} U_{k-s_{p}-1,s_{p}}\\ Y_{k-s_{p}-1,s_{p}} \end{array}\right]. \end{array}$$

where $Z_{f}$ and $Z_{p}$ represent future and past collected data, respectively. Therefore, LQ decomposition is performed on the data set

(21) $$\begin{array}{c} \left[\begin{array}{c} Z_{p}\\ U_{k,s}\\ Y_{k,s} \end{array}\right]=\left[\begin{array}{ccc} L_{11} & 0 & 0\\ L_{21} & L_{22} & 0\\ L_{31} & L_{32} & L_{33} \end{array}\right]\left[\begin{array}{c} Q_{1}\\ Q_{2}\\ Q_{3} \end{array}\right], \end{array}$$

where

(22) $$\begin{array}{c} \left[\begin{array}{c} Q_{1}\\ Q_{2}\\ Q_{3} \end{array}\right]\left[\begin{array}{ccc} Q_{1}^{T} & Q_{2}^{T} & Q_{3}^{T} \end{array}\right]=\left[\begin{array}{ccc} I & 0 & 0\\ 0 & I & 0\\ 0 & 0 & I \end{array}\right]. \end{array}$$

Finally, $U_{k,s}$ and $Y_{k,s}$ is expressed as

(23) $$\begin{array}{c} Z_{f}{\left[\begin{array}{c} Z_{p}\\ U_{k,s} \end{array}\right]}^{T}=\left[\begin{array}{cc} L_{21} & L_{22}\\ L_{31} & L_{32} \end{array}\right]{\left[\begin{array}{cc} L_{11} & 0\\ L_{21} & L_{22} \end{array}\right]}^{T}, \end{array}$$

(24) $$\begin{array}{c} Y_{k,s}=\left[\begin{array}{cc} L_{31} & L_{32} \end{array}\right]\left[\begin{array}{c} Q_{1}\\ Q_{2} \end{array}\right]+L_{33}Q_{3}. \end{array}$$

In a word, the dynamic method maps the input and output data into several stacking matrices to establish the system model. In addition, the high-speed train is vulnerable to external interference and condition switching, such as acceleration and deceleration. Therefore, the internal parameters change in real time, so that the dynamic method is more appropriate in electric drive systems. Compared with static methods, it is often not rigorous enough to deal with such situations.

#### 4.1.2. Definition of Test Statistics

Based on the obtained features of signals, two test statistics, the $T^{2}$ and squared prediction error (SPE), are defined at *k*-th time instance as follows

(25) $$\begin{array}{cc} T^{2}\left(k\right) & =z^{T}\left(k\right)P_{p}\Lambda_{p}^{-1}P_{p}^{T}z\left(k\right)\\ \mathrm{SPE}(k) & =z^{T}\left(k\right)\left(I_{k_{z}\times k_{z}}-P_{p}P_{p}^{T}\right)z\left(k\right) \end{array}$$

Then a reliable fault detection task can be achieved according to the following binary decision

(26) $$\begin{array}{cc} \; & \mathrm{Fault}\mathrm{and}\mathrm{alarm}⟸T^{2}\left(k\right)\geq J_{th,T^{2}}\mathrm{or}\mathrm{SPE}\geq J_{th,\mathrm{SPE}}\\ \; & \mathrm{Fault-free}⟸\mathrm{otherwise} \end{array}$$

where $J_{th}$ signifies the threshold of test statistics. In practical scenarios, the flood of false alarms must be prohibited so that the detection results can provide the conductor an auxiliary and effective indicator. Therefore, determining proper thresholds is usually accomplished via abundant tests [6]. It should be pointed out that the distribution of test statistics, such as the Chi-square distribution, cannot be directly used as a look-up table because z(k) is non-Gaussian.

### 4.2. Fault Diagnosis

In addition, taking PCA as an example, $\hat{z}\left(k\right)$ and $\tilde{z}\left(k\right)$ under faulty cases will be

(27) $$\begin{array}{c} {\hat{z}}^{f}\left(k\right)=\hat{z}\left(k\right)+P_{p}P_{p}^{T}\Omega_{i}f_{i}\left(k\right),\\ {\tilde{z}}^{f}\left(k\right)=\tilde{z}\left(k\right)+P_{r}P_{r}^{T}\Omega_{i}f_{i}\left(k\right). \end{array}$$

As shown in (27), the necessary condition that $f_{i}$ can be detectable and diagnosable is

(28) $$PP^{T}⊬\Omega_{i}$$

where ⊬ denotes the non-orthogonality between two spaces. After the fault being detected successfully, then the objective of fault diagnosis is

(29) $$\max\;\mathrm{Pr}(f\in f_{i}|PP^{T}\Omega_{i}f),$$

or

(30) $$\max\;\mathrm{Pr}(f\in f_{i}|\Omega_{i}f)$$

where Pr(·) is the probability.

In fact, the implementation of (29) is depended upon the fault feature; whilst the formulation of (30) is directly based on fault information hidden in original signals.

### 4.3. Comprehensive Evaluation Indices

At present, comprehensive evaluation indexes of FD can be considered from two dimensions in electrical drive systems: (1) the actual engineering indexes are analyzed to understand the engineering requirements; (2) the differences of performance indexes among the methods are verified in the laboratory. The following two dimensions are introduced in detail.

In practical engineering, FD is also called quality evaluation in electrical drive systems. The main method of quality evaluation is to determine the fault level by the score of the evaluation. Specifically, it is divided into the following steps:

Step 1: Standards of fault deduction. The identified faults include four types: A, B, C and D. Among them, 5 points will be deducted for class A, 10 points for class B, 20 points for class C and 100 points for class D. Partial score standards of faults of electrical drive systems are listed in Table 2.

Step 2: According to Table 2, the quality score of electrical drive systems can be given

(31) Truescore=1000−Faultscore×8.

where the true score must not be negative, and the full score for quality is 1000 points.

Step 3: Based on the true score, the evaluation list of electrical drive systems is presented in Table 3.

Step 4: Finally, engineers repair the damaged parts according to the true score.

The above steps can effectively provide more direct maintenance for electrical drive systems in the actual project, which is helpful to help engineers identify different fault levels. In addition, in order to further measure the superior performance of different algorithms, the following comprehensive evaluation indicators of false alarm rate (FAR) and missing alarm rate (MAR) are given in the laboratory.

FAR is defined as the probability that normal operations are judged to be faulty, which can be expressed as

(32) $$\mathrm{FAR}=P(J>J_{th}|f=0),$$

where *J* is the test statistic or evaluation function to measure the system. Generally, there are different definitions of *J* about the requirements of the system [35]. For example, *J* has $\ell_{2}$-norm, root square mean, $\ell_{\infty }$-norm and other forms to provide a more accurate measurement standard. $J_{th}$ is represented as a threshold. The setting of $J_{th}$ is directly related to *J*. In fault detection, FAR has important physical meaning to some extent.

MAR is defined as the probability that faults are not successfully detected, which can be expressed as

(33) $$\mathrm{MAR}=P(J<J_{th}|f\neq 0).$$

Compared with FAR, MAR is concerned with the fault detection ability of the system in case of fault.

Hence, it is helpful to the scientific research and engineering application of HIL to select the appropriate evaluation indices based on the actual engineering needs.

### 4.4. An Overview of FD Methods

The HIL-based platform has been widely used by a large number of experts and scholars to verify the effectiveness of the proposed FD methods [36,37,38]. From the perspective of model-based diagnosis method, a real-time FD method for sensors and IGBTs of the impulse rectifier is proposed in [39]. It is based on the structural analysis of electrical drive systems whose structural model has been established. The structural model under various fault conditions is evaluated and optimized. In [11], an FI strategy for safety testing and FD verification is presented in the electrical drive control systems. By simulating fault scenarios, the influence of fault signals on the system is analyzed from the perspective of mechanism. The FI strategy based on signal adjustment is adopted to pave the way for subsequent data-driven FD experiments.

As a common abnormality of the electrical drive systems, the incipient fault has always been an international problem that needs to be solved urgently. Since it is hard to identify the existence of incipient faults from the perspective of model-based diagnosis method, data-driven fault diagnosis method has been widely concerned, and a large number of research results are published [40,41]. Aiming at the crowding problem caused by incipient fault, a fault detection and diagnosis method is proposed based on probability-relevant PCA [25]. The effectiveness of the proposed method is verified in incipient FD by using HIL platform. In [42], considering the heterogeneity of sensor distribution, a multi-blocks system monitoring scheme is proposed. In the experimental verification, HIL is used to compare the performance on the different comprehensive evaluation indexes. For multimode fault detection, a just-in-time-learning aided CCA is proposed in [31] by using the system modal change of HIL platform, which overcomes the traditional method in the single working condition process.

In summary, the HIL platform provides an integration of environment of FD and FD, which improves the possibility for the practical application of advanced theoretical methods.

## 5. Conclusions

In this paper, an HIL-based platform has been developed for simulating the realistic faults in electrical drive systems of high-speed trains. Then FD algorithms have been tested to detect the obtained faults, whose performance can be evaluated via comprehensive indices. This platform is an integrated design that covers both FI and FD techniques, providing engineers or researchers with a reliable simulation system. The design platform is developed based on an electrical drive system of CRH2-type trains. The abundant attempts of FD have illustrated its effectiveness and feasibility. In the coming ten years, the authors believe that, based on this tutorial, there will be lots of follow-up publications and applications. It can not only analyze the electrical fault of the electric drive system but also identify the mechanical fault.

## Figures and Tables

**Table 1:** Key signal features and data processing approaches.

Features	Approaches
Mean	Expectation operation
Variance/covariance	Principal component analysis
High-order statistics	Independent component analysis
Correlation	Canonical correlation analysis
Slope	First-order difference
Entropy	Mutual information
Waveform index	Wavelet transform
Root mean square	Partial least squares

**Table 2:** Evaluation standards of electrical drive systems.

Standards of Quality	Faults Types			
	A	B	C	D
Main transformer and its cooling	Poor cleaning	Loose wiring, and invalid desiccant	Abnormal fan rotation and low liquid level or oil spill	Traction converter faults
Traction converter and its control	Poor cleaning	Loose wiring, and invalid desiccant	Abnormal fan rotation and traction converter faults	None
Traction motor and its cooling	Poor installation of oil filling plug and unstable cooling duct	Loose fittings, broken wiring, missing oil injection plug and blocked exhaust	Loose and damaged power line, sensor and wiring	Cracked mount and bad motor
Auxiliary converter	None	None	One dysfunction	Two dysfunctions

**Table 3:** Fault level results of electrical drive systems.

Fault Levels	True Scores
A	900–1000
B	800–899
C	700–799
D	699 or less

## Data Availability

Not applicable.
